# Microbiota-derived proteins synergize with IL-23 to drive IL22 production in model type 3 innate lymphoid cells

**DOI:** 10.1371/journal.pone.0317248

**Published:** 2025-01-13

**Authors:** Yanling Wang, David S. J. Allan, Andrew T. Gewirtz

**Affiliations:** 1 Center for Inflammation, Immunity, & Infection, Institute for Biomedical Sciences, Georgia State University, Atlanta, Georgia, United States of America; 2 Cellular and Molecular Therapeutics Branch, National Heart Lung and Blood Institute, National Institutes of Health, Bethesda, Maryland, United States of America; Kansai Medical University: Kansai Ika Daigaku, Institute of Biomedical Science, JAPAN

## Abstract

Microbiota-induced production of IL-22 by type 3 innate lymphoid cells (ILC3) plays an important role in maintaining intestinal health. Such IL-22 production is driven, in part, by IL-23 produced by gut myeloid cells that have sensed select microbial-derived mediators. The extent to which ILC3 can directly respond to microbial metabolites via IL-22 production is less clear, in part due to the difficulty of isolating and maintaining sufficient numbers of viable ILC3 *ex vivo*. Hence, we, herein, examined the response of the ILC3 cell line, MNK-3, to microbial metabolites *in vitro*. We observed that fecal supernatants (FS), by themselves, elicited modest levels of IL-22 and synergized with IL-23 to drive robust IL-22 production assayed by qRT-PCR and ELISA. The IL-22 synergistic activity of FS was not mimicked by an array of candidate microbial metabolites but could be attributed to bacterial proteins. Examining how MNK3 cells exposed to IL-23, FS, both, or neither via RNA-seq and immunoblotting indicated that FS activated MNK-3 cells in a distinct pattern from IL-23: FS activates p-38 MAPK while IL-23 activates STAT3 signaling pathways. Collectively, these studies indicate ILC3 sensing of microbiota proteins promotes IL-22 production suggesting the possibility of manipulating microbiota to increase IL-22 without risk of IL-23-mediated chronic inflammatory diseases.

## Introduction

IL-22 plays a key role in the maintenance of epithelial, and consequently mucosal health. IL-22 drives mucus production by goblet cells and promotes secretion of antimicrobial peptides and proliferation by epithelial cells [1]. Deficiency of IL-22 promotes infectious and idiopathic colitis, obesity, and metabolic syndrome. IL-22 can be produced by a range of leukocytes including CD4 T cells (Th17, Th22), γδ T cells, neutrophils and type 3 innate lymphoid cells (ILC3). The relative contributions of these cell types to the total production of IL-22 in an unmanipulated wild type host is unclear but, at least in host lacking CD4 T-cells, ILC3-mediated IL-22 is important in that its absence results in systemic inflammation due to inability to contain commensal bacteria [2, 3]. ILC3-mediated IL-22 production has been studied *ex vivo* using preparations of intestinal cells, particularly Peyer’s Patch cells (PPC), which contain a variety of cell types and thus permit paracrine signaling thought to be critical for IL-22 production. Indeed, the best characterized means of ILC3-mediated IL-22 production is that driven by IL-23, and to a lesser extent IL-1β, both of which can be produced by intestinal phagocytes exposed to select stimuli, including the bacterial protein flagellin via activation of TLR5 and NLRC4, respectively. Yet, the relatively normal regulation of IL-22 expression in mice lacking TLR5 and NLRC4 in scenarios not involving exogenous flagellin administration [4] suggest that numerous other drivers of IL-22 expression have yet to be defined.

The stark reduction in IL-22 levels in germfree mice suggests that a predominant role for microbial-derived metabolites in driving its expression. Indeed, we recently reported that fecal supernatant activates IL-22 in ILC3 in cultured Peyer’s Patches with outer membrane protein A (OmpA) of gut commensal bacteria *Parabacteroides goldsteinii*, present in fecal supernatants (FS) of mice fed compositionally defined diets enriched with fermentable fiber, inulin, being one specific metabolite capable of partially recapitulating this bioactivity [5]. In contrast to flagellin, elicitation of this IL-22 production from ILC3 *ex vivo* was independent of IL-23, potentially reflecting it was driven by other phagocyte-produced paracrine mediators and/or that ILC3 themselves were directly responding to this bacterial protein. Addressing this specific question, and more generally the extent to which ILC3 can directly sense products produced by the microbes they help contain, has been stymied by the difficulty of isolating and maintaining sufficient numbers of viable ILC3 *ex vivo*. Herein, we surmounted this problem by utilizing a recently developed immortalized ILC3 cell line, namely MNK-3 [6]. We found that MNK-3 did not respond to OmpA but did respond to yet to be fully identified microbial products present in FS and did so in a manner that was distinct from, and synergistic with IL-23.

## Materials and methods

### Cell culture

MNK-3 cell line [6] was maintained in DMEM supplemented with 10% heat-activated FBS, 2mM GlutaMAX, 1 mM Sodium pyruvate, 55μM 2-mercaptoethanol, 10 mM HEPES, 50 μg/ml gentamycin, 100 U/mL penicillin and streptomycin and 10 ng/mL recombinant mIL-7 and mIL-15.

### Mice

8-week-old female C57BL/6 mice were purchased from Jackson Labs (Bar Harbor, ME) and maintained at Georgia State University via procedures approved by its institutional animal use and care committee under protocol #s A17047 and A24001. Mice were fed compositionally defined diets enriched with inulin(200g inulin per 4047 kcals, Research Diet #D13081108) for a duration of 4–6 weeks and the resulting fecal supernatant was referred as “FS”. Feces were collected daily starting from one week on diet and stored at −80°C for later analysis. Antibiotics, when indicated, containing vancomycin (0.5 g/L), neomycin (1 g/L), metronidazole (1 g/L) and ampicillin (1 g/L) was given to mice in drinking water for 2 weeks while mice remained on inulin diet. Feces were collected starting from one week following antibiotic treatment and stored at −80°C for later analysis.

When specified, mice were fed a cellulose-enriched diet (200g cellulose per 4047 kcals, Research Diet #D13081109) and the fecal supernatant was referred as “FS-Cel”. Germ-free mice were fed a standard grain-based chow diet and the fecal supernatant was referred as “FS-GF”.

### Preparation of fecal supernatant (FS) and its OMVs

Feces were collected according to the above methods and were resuspended in PBS as 100 mg/mL and vortexed extensively until no visible pellet was seen, prior to centrifugation (21,000 x g for 10min with the exception of 40 min for inulin feces). Supernatant (FS) was aliquoted and stored at −80°C. For OMV preparation, FS was further ultra-centrifuged at 110,000 x g at 4°C or 2 h. The pellet (OMV) was suspended and sonicated briefly in PBS.

### ELISA

MNK-3 cells were seeded in 96-well plate, with a density of 20,000 cells in 100 μL media, with or without 10 ng/μL recombinant mIL-23 primed. 20 μL of FS-Inu or PBS was added to the cells 2–3 hours afterwards. Culture supernatant was harvested after 22 hours of incubation. IL-22 production was measured by DuoSet Mouse IL-22 ELISA kit (R&D) according to the manufacture’s instructions.

### RNA isolation and RT-qPCR

MNK-3 cells were seeded in 12-well plate, with a density of 300,000 cells in 600 μL media, with or without 10 ng/μL recombinant mIL-23 primed. FS (150 μL) or PBS was added to the cell 18–20 hours afterwards. Total RNA was isolated from cells 6 hours or indicated time after incubation, using RNeasy Plus Mini kit (Qiagen).

Quantitative RT-PCR was performed using the iScript One-Step RT-PCR Kit with SYBR Green (Bio-Rad, Hercules, Californai) in a CFX96 apparatus (Bio-Rad, Hercules, California) with the primers in the following: IL-22 5L-22 tative RT-PCR was pe and 5′-TGGATGTTCTGGTCGTCACC-3′; 36B4 5′-TCCAGGCTTTGGGCATCA-3′ and 5′-CTTTATTCAGCTGCACATCACTCAGA-3′. Differences in transcript levels were quantified by normalization of each amplicon to housekeeping gene 36B4 and standardized to PBS.

### RNA-seq

RNAseq library was prepared and sequenced at Molecular Evolution Core of Georgia Institute of Technology on NextSeq (Illumina, 1x 75bp). 40-50M reads were obtained per sample. Data analysis was done on Galaxy. Briefly, reads were trimmed from 3’ end at a quality cutoff at 20 using Cutadap, following mapping against mouse genome (GRCm38, version M25) using STAR. Number of reads per gene were quantified by FeatureCounts and normalized across samples using DEseq2. Differentially expressed genes between FS-Inu or IL-23 or the combination versus PBS (p-adj<0.05 and fold change>2) generated by DEseq2 were analyzed for KEGG pathway enrichment analysis using Goseq. Normalized counts data were processed and visualized by heatmap and PCA plot by R (4.2.3). RNA-seq data was deposited in NCBI SRA (Sequence Read Archive), PRJNA1162128.

### Western blot

MNK-3 cells were seeded in 6-well plates, with a density of 1 million cells in 2 mL media. 520 μL of FS-Inu or PBS or mIL-23 (10 ng/μL) or the combination was added to the cells and incubated for 15min, 1 hour, 2hours or 3 hours. Cells were then lysed in 200 μL RIPA supplemented with protease inhibitor and phosphatase inhibitor. Protein concentration in the cell lysate were quantified by BCA protein assay (Pierce^™^ Rapid Gold BCA Protein Assay Kit, Thermo Scientific). 4 μg of total protein per sample were separated by SDS-PAGE on a 4–20% gradient mini precast gel (Bio-Rad). Proteins were transferred to PVDF using Trans-Blot Turbo Transfer System (Bio-Rad). After blocking in 5% skim milk, membranes were incubated with primary antibody (Table 1) for overnight at 4°C. Blots were washed and incubated with secondary antibody (anti-rabbit, Cell Signaling) for 1 hour at room temperature and developed using ECL substrate high sensitivity kit (Abcam) and imaged by Chemidoc (Bio-Rad). Blots in Figs 5C and S3B were stripped (1.5% glycine, 0.1% SDS, 1% Tween-20, pH = 2.2) for 15 min, reblocked and reprobed for actin. The intensity of bands in S3B Fig was quantified using Image Lab (Bio-Rad).

**Table 1:** Western blotting antibodies.

Antigen	Predicted Molecular Weight (kDa)	Cell Signaling Catalog number
t-STAT3	79,86	12640S
Phosphorylated STAT3 (p-STAT3)	79,86	9145S
t-P38	40	8690S
Phosphorylated p38 (p-p38)	43	4511S
t-Erk	42/44	4695
Phosphorylated Erk (p-ErK)	42/44	4370S
t-c-Jun	48	9165S
Phosphorylated c-Jun (p-c-Jun)	48	3270S
Actin	45	4970S

### Data analysis and statistics

Values are expressed as mean ± SEM. Statistically difference was determined by unpaired two-tailed t test, or one-way ANOVA followed by Dunnett’s multiple comparisons in Graphpad Prism. For RNA-seq data, data was analyzed by Galaxy and R. *,**,***,**** indicate p<0.05, 0.01, 0.001, 0.0001, respectively.

## Results

### Fecal supernatant and IL-23 synergized to drive IL-22 production from MNK-3 cells

**Fig 1:**
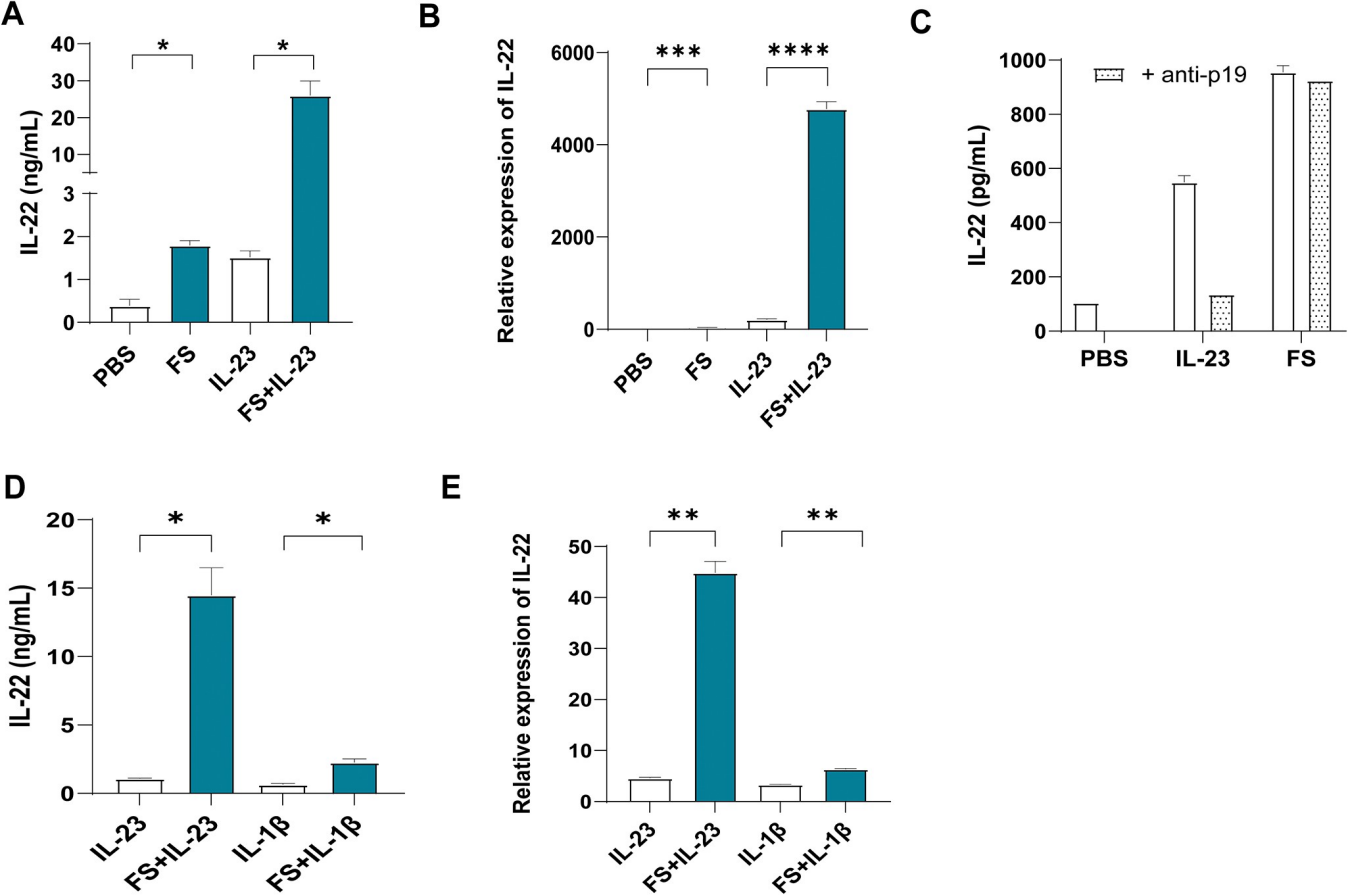
Fecal supernatant and IL-23 synergized to drive IL-22 production from MNK-3 cells. (A and B) IL-22 production of MNK3 cells in response to FS (1.7% m/v), IL-23 (10 ng/mL), both or neither, assayed by ELISA (A) and qPCR (B). (C) IL-22 production of MNK3 cells in response to IL-23 or FS, with or without supplementation of anti-IL-23 p19 neutralizing antibody, assayed by ELISA. (D and E) IL-22 production of MNK3 cells in response to IL-23 or IL-1β alone, or the combination of FS and IL-23 or IL-1β, assayed by ELISA (D) and qPCR (E). Data are means ± SEM. *,**,***,**** indicate p<0.05, 0.01, 0.001, 0.0001, respectively, by unpaired two-tailed t tests.

MNK-3 cells, cultured in 10% FBS, basally produced IL-22 at levels that were modest but nonetheless readily detectable by ELISA. Such IL-22 production was elevated about 4-fold in response to a 24-h exposure to fecal supernatant (FS) (Fig 1A), which was isolated from mice fed a fiber rich diet, which promotes IL-22 expression *in vivo* [7]. A similar increase in IL-22 production was observed in MNK-3 cells exposed to IL-23, which was administered at levels previously used to induce IL-22 in primary ILC3. The combination of FS and IL-23 resulted in a 65-fold induction of IL-22 production indicating synergy between these agonists. Elevations in production of IL-22 in response to FS, IL-23, and the combination thereof, were paralleled by similar changes in IL-22 mRNA levels (Fig 1B). FS-induced IL-22 production was IL-23-independent in that it was not reduced by IL-23 neutralization (Fig 1C). We investigated whether this FS might also synergize IL-1β, which also activates ILC3 to produce IL-22 [8, 9]. The combination of FS and IL-1β induced only modest elevations in IL-22 expression at the protein and mRNA level (Fig 1D and 1E), suggesting that ability to synergistically elevate IL-22 expression in these cells may be unique to FS and IL-23.

### FS activation of IL-22 is heat- and protease-sensitive and present in OMV

**Fig 2:**
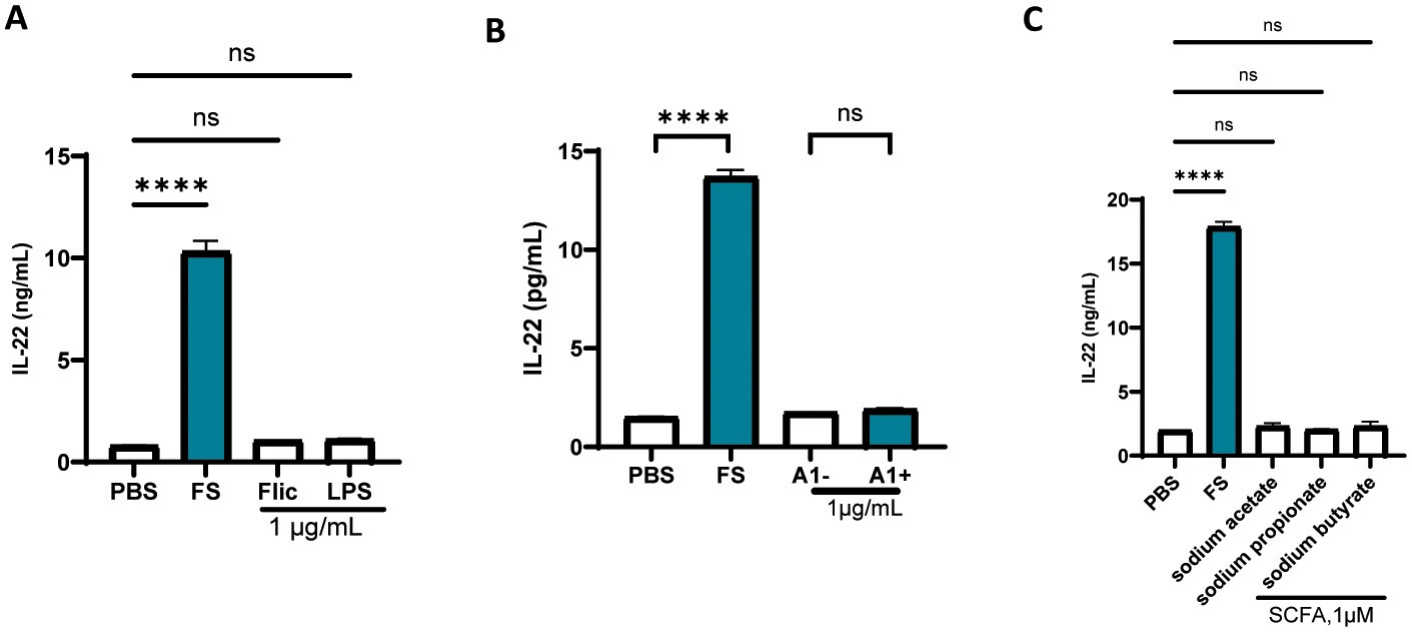
Flagellin, LPS, OmpA and SCFA did not promote IL-22 induction. IL-22 production of MNK3 cells (primed with IL-23, 10 ng/mL) in response to flagellin (flic, A), LPS (A), OmpA (B) and SCFA (C). Data are means ± SEM. ns, **** indicate p>0.05, and <0.0001, respectively, by one-way ANOVA, followed by Dunnett’s multiple comparisons to PBS (A and C) and by two-tailed unpaired t test (B).

**Fig 3:**
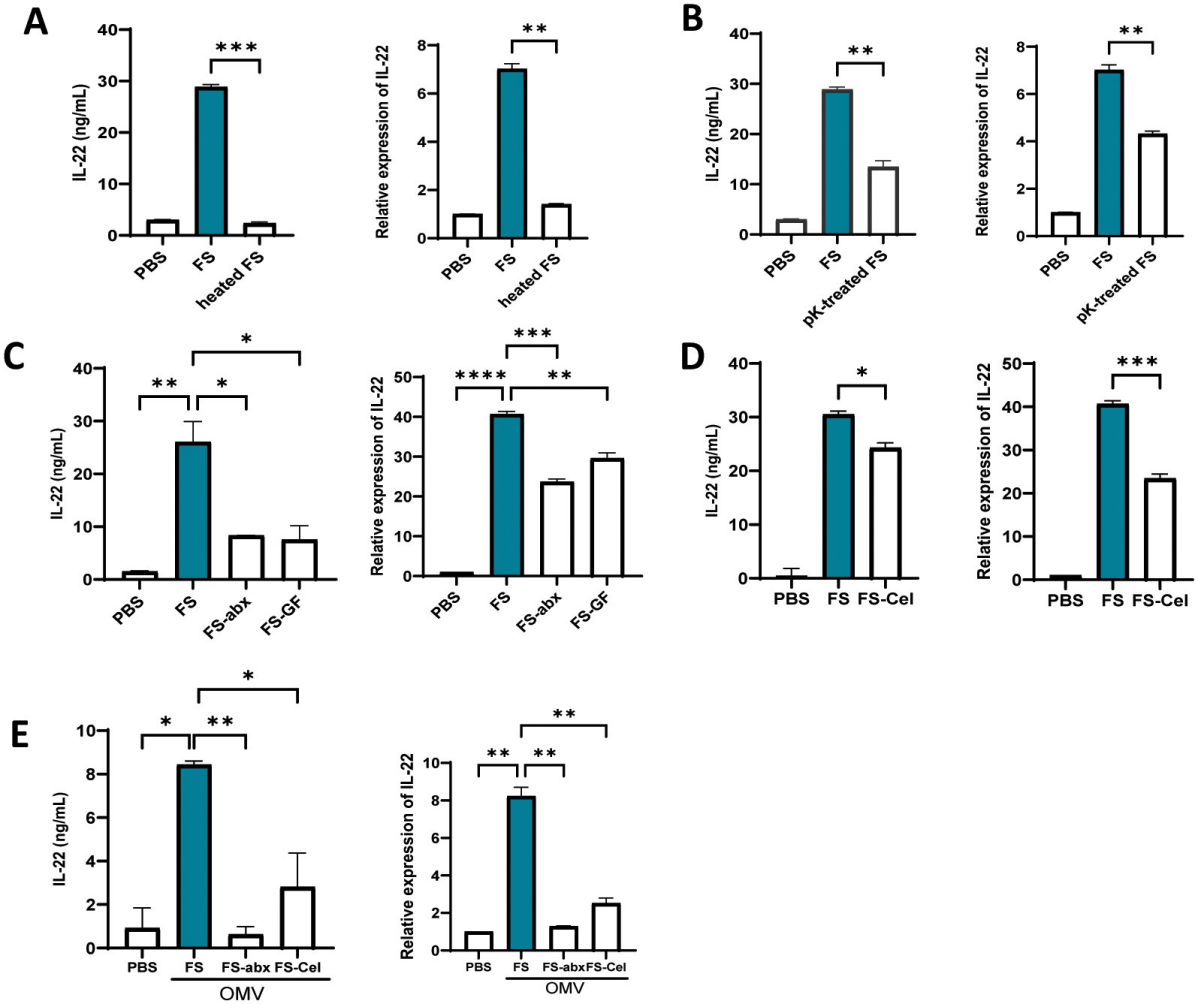
FS’ IL-22 promoting activity is heat- and protease-sensitive and present in OMV. IL-22 production of MNK3 cells (primed with IL-23, 10 ng/mL) in response to FS heated at 90 °C for 20 minutes (A), treated with agarose beads-linked proteinase K overnight (B), FS of mice with antibiotic cocktails in the drinking water (FS-abx) or germ-free mice (FS-GF) (C), FS of mice fed with cellulose diet (FS-Cel) (D), outer membrane vesicles isolated from FS, FS-abx or FS-Cel (E). Data are means ± SEM. *,**,***,**** indicate p<0.05, 0.01, 0.001 or 0.0001, respectively, by one-way ANOVA, followed by Dunnett’s multiple comparisons to FS (C and E), two-tailed unpaired t tests (A, B and D).

We next sought to identify the component of FS that promoted IL-22 production by MNK-3 cells. First, we considered 2 molecules, namely flagellin and OmpA, that, when administered to PPC *ex vivo*, can increase IL-22 production by ILC3 [5, 10]. Neither molecule increased MNK-3 IL-22 production irrespective of IL-23 priming (Fig 2A and 2B). Short-chain fatty acids (SCFA), which are produced in abundance by fiber-fed hosts are reported to promote IL-22 expression but, in contrast to FS, neither butyrate, propionate, nor acetate did so in IL-23-primed MNK-3 cells (Fig 2C). These results argued against a role for these candidate mediators and thus promoted us to use more general biochemical approaches. We found that heating FS to 90°C eliminated its ability to promote IL-22 expression at the protein and mRNA levels (Fig 3A). Assay of MNK-3 expression by ELISA and q-RT-PCR also found that FS’ IL-22 promoting activity was reduced, but not eliminated, by protease K treatment (Fig 3B). These results suggest the involvement of heat- and proteinase-labile compounds, likely proteins. We next investigated whether the IL-22 promoting activity of FS is bacterial derived. We isolated FS from germfree mice, which lack live bacteria in their feces (but contain some bacterial derived components in their sterilized diets), or mice administered antibiotics cocktail, which lowers bacterial loads by about 100-fold. Both approaches resulted in large reductions in FS’ IL-22 promoting activity (Fig 3C). We also measured this parameter in FS isolated from mice fed a diet lacking fermentable fiber (FS-Cel) and thus having a 10-fold reduction in fecal bacterial density. This resulted in a more modest reduction in FS’ IL-22 promoting activity (Fig 3D). Lastly, we examined if IL-22 inducing activity could be captured in outer membrane vesicles (OMVs), prepared from FS. OMVs are nanoparticles that are released from the outer membranes of bacteria and are increasingly appreciated to mediate microbiota influences on its host [11]. Indeed, we found that OMV from FS, but to a much less extent from FS-Cel, captured the IL-22 promoting activity and this was the case at both the protein and mRNA levels (Fig 3E). The complete loss of IL-22 promoting activity in OMV from FS-abx confirms that these OMVs were indeed derived from bacteria. Together, these results suggest that FS’ IL-22 promoting activity involved bacterial-derived heat-sensitive protein(s) that are secreted in OMV.

### FS and IL-23 activated MNK3 in distinct patterns

**Fig 4:**
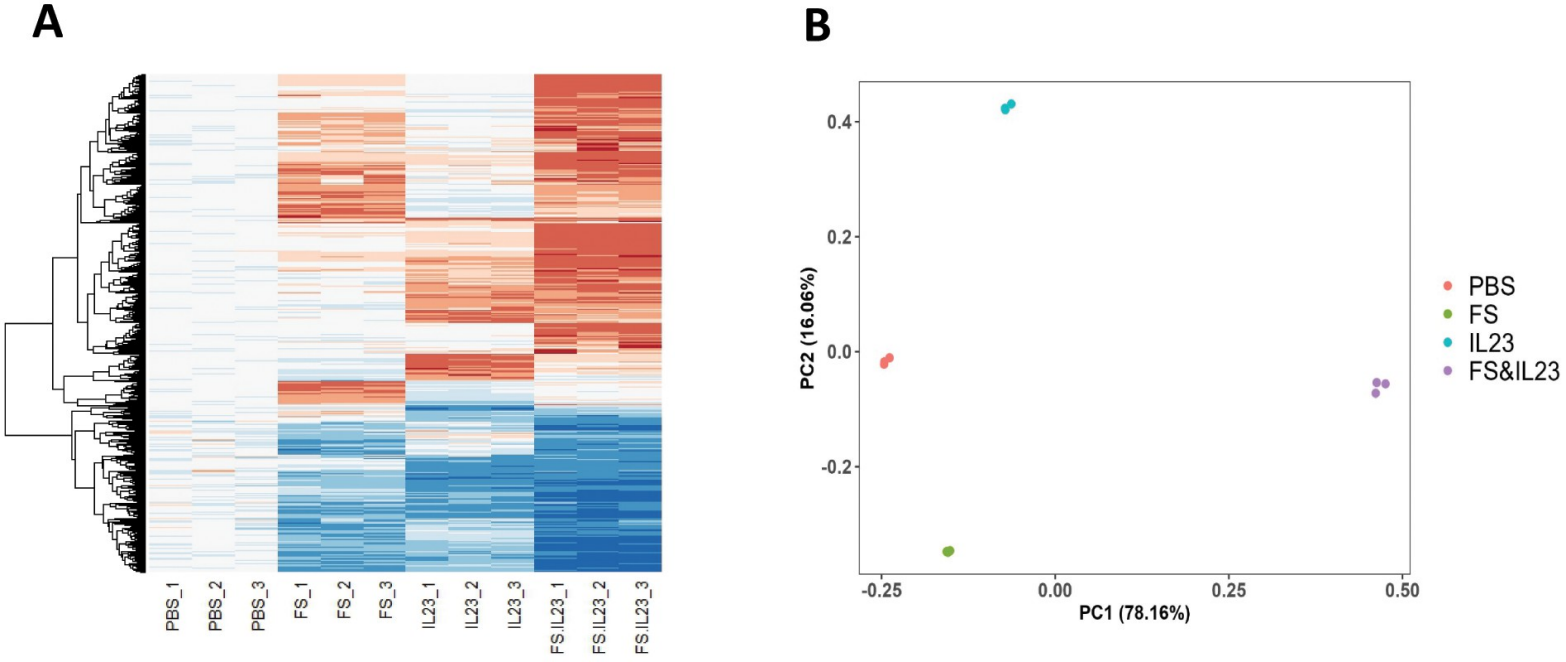
FS and IL-23 activated MNK3 in distinct patterns. MNK3 cells were treated with FS, IL-23, both or neither for 6 hours. RNA was isolated and sequenced. (A) Heatmap of genes significantly altered (p < 0.05) by 2-fold in any group relative to the PBS. Normalized gene counts data were transformed to z-score, and standardized by PBS (PBS as zero). (B) PCA plot.

We next wanted to probe if the remarkable synergism between FS and IL-23 in driving IL-22 production broadly extended to their impacts on MNK-3 gene expression. MNK-3 gene expression was profiled via RNA-seq 6 hours following exposure of these cells to PBS, FS, IL-23 or both (FS&IL-23), n = 3 replicate cell cultures per condition. Visualizing the results as a heat map, of all genes whose expression was altered by > 2-fold relative to PBS by any condition, reveals modest distinct impacts by FS or IL-23 by themselves and a much more pronounced alteration in gene expression by their combination (Figs 4A, S1A and S1B). Such combined impacts of FS and IL-23 largely reflected that genes whose expression was induced by either stimulus were induced to a greater extent by their combination although a modest subset of genes induced by either FS or IL-23 alone were not induced by their combination. In accord with this notion, principal component analysis (PCA), found clear separation of all 4 conditions with a particularly dramatic separation between FS&IL-23 and PBS along PC1 thus indicating that FS and IL-23 had broad synergistic impacts on MNK-3 gene expression relative to either stimulus by itself (Fig 4B). FS and IL-23 were largely separated along PC2 suggesting that, in addition to their synergistic interactions, each has a distinct impact on MNK-3 gene expression. PC loading analysis revealed that major drivers of changes along PC1 were *Il17f*, *Il22*, and *Il2ra*. while the largest influencers on PC2 were *Mt-Rnr1/2*, *Il2rb* and *Ermp1* on PC2 (S1C Fig). As PC1 explains 78% of the global gene variance, and IL-17 is often co-expressed with IL-22, this result indicates that the biggest overall transcriptional difference between FS&IL-23 treatment and PBS could be attributed to the expression of IL-22 and IL-17.

### FS activates p-38 MAPK while IL-23 activates STAT3 signaling pathways

**Fig 5:**
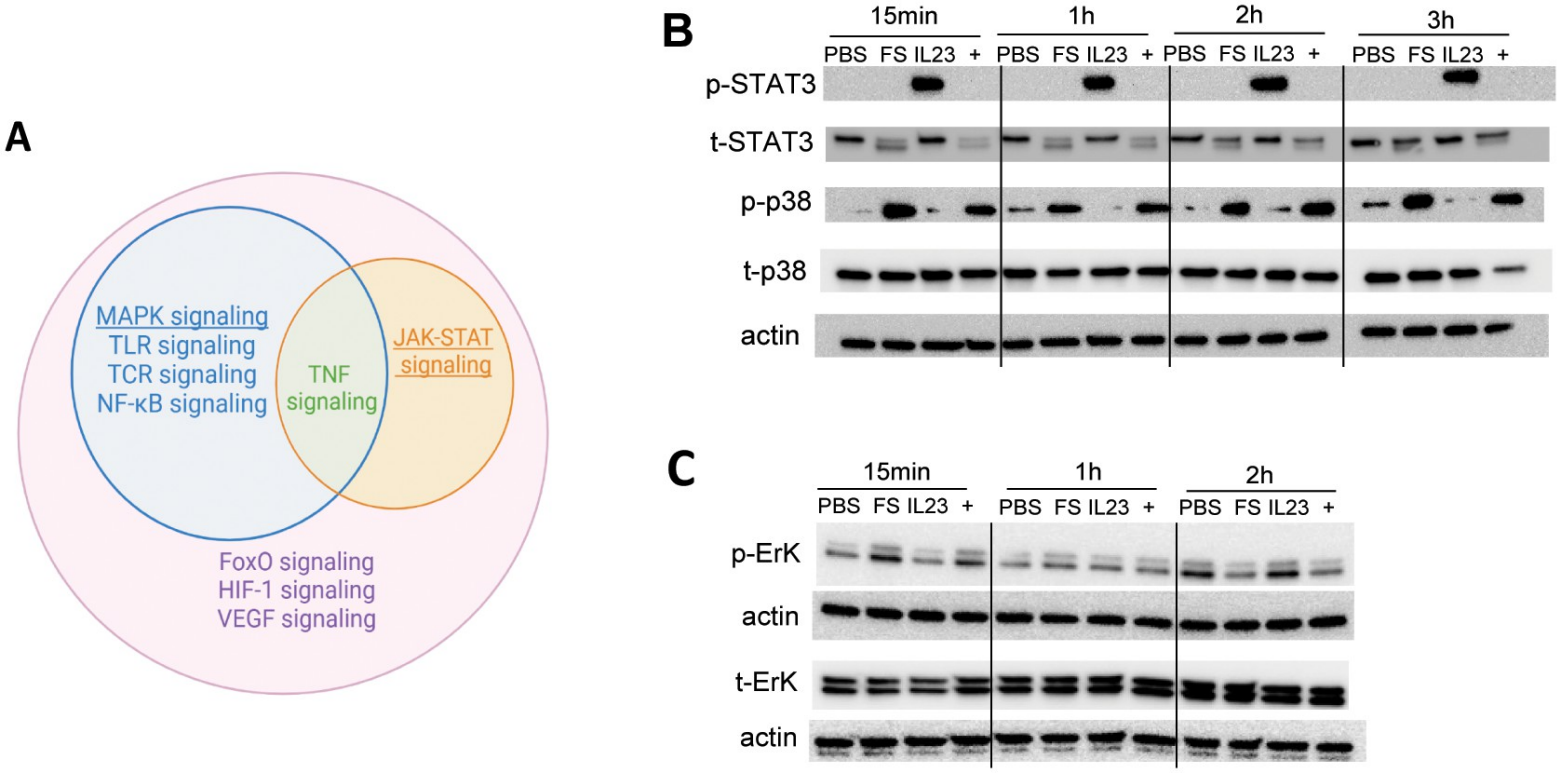
FS activates p-38 MAPK while IL-23 activates STAT3 signaling pathway. (A) Venn diagram of KEGG signaling pathways. Pathways contained “signaling” and are significantly different in indicated pairs (Benjamini-Hochberg adjusted p-values<0.10) are shown in the diagram. The outside pink circle represents signaling pathways differentially expressed between PBS vs combo; blue circle PBS vs FS; orange PBS vs IL-23. (B) Immunoblotting of STAT3, p38 and actin. (C) Immunoblotting of ErK. Blots of p-ErK and t-Erk were eacj stripped and reprobed for actin. See raw images and details of Fig 5B and 5C in S1 File.

We next sought to understand mechanisms that underlie the differential and synergistic impacts of FS, IL-23, and their combination on MNK-3 gene expression. The differentially expressed genes yielded by RNA-seq (adjusted p<0.05 and FC>2) were analyzed by Goseq for KEGG pathways. The results, displayed as a Venn diagram suggested that MAPK, Toll-like receptor, and NF-κB signaling pathways were uniquely influenced by FS, while the JAK-STAT signaling pathway was uniquely influenced by IL-23 (Fig 5A). MAPK signaling has been reported to regulate IL-22 expression in ILC3 [12] whereas JAK-STAT is the canonical pathway that mediates IL-23-induced signaling. We therefore examined the STAT3 and MAPKs (p38, JNK, and ERK) phosphorylation by SDS-PAGE immunoblot analysis. We found that IL-23 increased STAT3 phosphorylation in 15min and the activation sustained for 3h post stimulation (Fig 5B). There was no synergistic or even additive effect on phosphorylated STAT3 levels; rather, FS appeared to inhibit the activation of STAT3 by IL-23. Interestingly, FS transiently decreased total STAT3 levels (at 15min and 1 hour). Conversely similarly, FS increased p-38 phosphorylation in 15min and the activation sustained for 3h post stimulation (Fig 5B). In contrast IL-23 did not induce p38 MAPK phosphorylation nor did it impact that induced by FS. An analogous but less pronounced pattern of p-ERK induction was observed particularly at the 15 minutes time point (Fig 5C) while a clear pattern of changes in p-JNK levels was not observed (data not shown). Collectively, these results accorded with Goseq’s prediction that IL-23 activated JAK-STAT3 signaling while FS activated MAPK signaling.

### FS increased phosphorylation of c-Jun

As an alternate approach to understand how FS and IL-23 synergized in driving IL-22 expression, we examined our RNA-seq data for relative expression of the array of transcription factors (TFs), namely STAT3, STAT5, AhR, RORrt, HIF1a, T-bet, and AP-1 that have been reported to regulate IL-22 expressions [12–19]. We plotted expression of each (S2 Fig) and computed fold change (FC) and differences (Delta) of expression levels, between “combo” treatment and sum of “FS” and “IL-23” treatments; and considered a synergism in these TFs if their FCs are larger than 2 and Deltas are larger than 0. This approach identified AP-1 family (*Jun*, *Junb*, *Fosl2*) and *Stat5a* (S3A Fig). We next examined the c-Jun and STAT5 phosphorylation by SDS-PAGE immunoblotting. We observed an increase in phosphorylated and total c-Jun levels phosphorylation in response to FS that were further modestly elevated by the combination of FS and IL-23, particularly at the 3 h time point (S3B Fig). We did not observe changes in levels of phosphorylated or total STAT5. That evidence of additive activation of c-Jun signaling was only observed at the 3 h time point while activation of STAT3 by IL-23 as early as 15 min, prompted us to examine the kinetics of IL-22 mRNA expression induced by IL-23 and the combination. Indeed, we observed that, while levels of IL-22 mRNA elicited by IL-23 plateaued within 2 hours, those induced by FS&IL-23 were observed as late as 6 h post-stimulation. Thus, the delay of Jun signaling of the combination is consistent with the IL-22 expression.

## Discussion

IL-22 is known to play a central role in mucosal health, in part, by driving enterocyte proliferation and expression of antibacterial mediators. The stark reduction in IL-22 levels in both germfree and ILC3-deficient mice underscores the important role played by microbiota-mediated activation of ILC3 in driving expression of this cytokine. Yet, the microbial products that drive ILC3-mediated IL-22 production have not been well defined. Nor is it clear if ILC3, themselves, directly sense microbial products to regulate IL-22 expression. Indeed, the best-defined microbial driver of ILC3-mediated IL-22 production, namely flagellin inducing dendritic cells to produce IL-23, which then acts on ILC3 to drive IL-22 expression, is not critical for homeostatic IL-22 production in that basal IL-22 levels are not reduced in TLR5-deficient mice [4]. One reason that ILC3 activators are poorly characterized is the difficulty of isolating and maintaining these cells in a sufficient quantity and a viable state *ex vivo*. The development of the MNK-3 cells as an ILC3 cell line offers a means to circumvent this hurdle. Like native ILC3, MNK-3 phenocopy the ability of ILC3 to produce IL-22 in response to IL-23. Herein, we used this tool to investigate the question of whether ILC3-mediated production of IL-22 might be regulated by these cells directly sensing bacterial products. We found that, indeed, MNK-3 cells produced IL-22 in response to microbiota-dependent protein(s) by a pathway that was both independent of, and yet synergistic with that elicited by IL-23. Activated ILC3 can also produce granulocyte-macrophage colony-stimulating factors (GM-CSF) and IL-17 [8]. We observed MNK-3 cells expressed IL-17 (*Il17f*) in response to FS and IL-23, in a synergistic way similar to IL-22 expression while they exhibited greater expression of GM-CSF (*Csf2*) in response to FS alone (S1A Fig). These results argue that ILC3 phenotype, including but not limited to IL-22 production, is not purely dictated by intestinal phagocytes. Rather, it suggests that ILC3 themselves sense bacterial products and, consequently, autonomously modulate their gene expression.

The specific protein(s) present in FS that promote MNK-3 cell IL-22 production remain to be identified. We expect that chromatographically fractionating FS, followed by testing fractions for IL-22 inducing bioactivity and, subsequently mass spectrometry will answer this question. We recently utilized this approach to discover that outer membrane protein A (OmpA) is one protein present in FS that drives IL-22 production from Peyer’s Path cells *ex vivo* [5]. Accordingly, we hypothesized that OmpA might drive IL-22 production by MNK-3 cells but did not observe this to be the case. We speculate this reflects that, like flagellin, OmpA does not directly activate ILC3 but rather acts on phagocytes present in Peyer’s Patch cells. In any case, while identification of unknown proteins based on a bioactivity readout is inherently challenging, we submit that extended use of MNK-3 cells should help meet this challenge although demonstrating that such molecules play a biologically significant role will require in vivo studies.

One of the most striking aspects of FS-induced activation of MNK-3 cells is that it induces a pattern of gene expression quite distinct from that induced by IL-23. Indeed, while our initial gene of interest, IL-22 was induced by both IL-23 and FS, an overall assessment of the transcriptomes of FS- and IL-23-stimulated MNK-3 cells found completely distinct sets of genes were differentially expressed in response to these stimuli. Nonetheless, this global assessment also deemed the stimuli to be synergistic in that most genes induced by either stimulus alone was induced much more so by their combination, although there were small clusters of genes that followed the opposite pattern (induced less by combination than either stimulus alone, Fig 4A). A considerable set of genes of which the expression was distinct by induction of FS and IL-23 may reflect that FS but not IL-23 activated MAPK signaling, especially p38 MAPK while IL-23 but not FS activated the STAT3 pathway. Potential drivers of the synergy between FS and IL-23 in activating expression of IL-22 and many other genes are less clear in that FS appeared to block STAT3 arguing against a role for this pathway. Our data accorded with a potential for the c-Jun/AP-1 pathway and thus recent findings that p38, also efficiently phosphorylates c-Jun at Ser-63 and Ser-73 and thus modulates the transcriptional activity of AP-1, in both CD4^+^ and CD8^+^ T-cells [20]. Discerning its role and how it might be activated by these stimuli will require further experimentation. Nonetheless, the distinct changes in signaling molecules and gene expression by FS and IL-23 and the synergistic changes in gene expression from their combination support the notion that ILC3s integrate sensing of host and microbial metabolites to maintain intestinal health. Moreover, these results support the use of MNK-3 cells to better understand the host-microbe interrelationship.

## Supporting information

S1 Fig FS and IL-23 activate MNK3 in distinct patterns. S1 Fig is related to Fig 4. (A and B). Top genes that were differentially expressed by FS (A) and IL-23 (B) compared to the control PBS (FC>8). (C). PCA loading analysis. *Il17f*, *Il22*, *Il2ra* had largest influences on PC1. *Mt-Rnr1/2*, *Il2rb*, and *Ermp1* had largest influences on PC2.
(TIF)

S2 Fig Gene expression of common transcription factors of IL-22. Normalized gene counts data was generated from RNA-seq data.
(TIF)

S3 Fig FS increased phosphorylation of c-jun. (A) Gene expression of common transcription factors (TF) of IL-22. Normalized gene counts of groups FS, IL23 and FS&IL23 were corrected by the background PBS. FC was the fold change of FS&IL23 to the sum of FS and IL-23 (FS&IL23/(FS+IL23)). Delta was the count difference between FS&IL23 and sum of FS and IL-23 (FS&IL23-(FS+IL23)). Genes were colored red if FC>2.0 and delta>0; otherwise blue. (B) Immunoblotting of c-Jun. Blots of p-c-Jun and t-c-Jun were each stripped and reprobed for actin. Band intensity was quantified by Image Lab (Bio-Rad). (C) IL-22 mRNA expression of MNK-3 cells, treated by IL-23 and FS&IL-23 for indicated time (2 hours, 6 hours, and 24 hours). See raw images and details of S3B an S3C Fig in S1 File.
(TIF)

S1 File Raw images of Figs 5B, 5C and S3B. (PDF)
