# Supplementary figures and images for: Microbiota-derived proteins synergize with IL-23 to drive IL22 production in model type 3 innate lymphoid cells
Source: PLoS One. 2025 Jan 13;20(1):e0317248. doi: 10.1371/journal.pone.0317248 (PMC11729933; doi:10.1371/journal.pone.0317248)

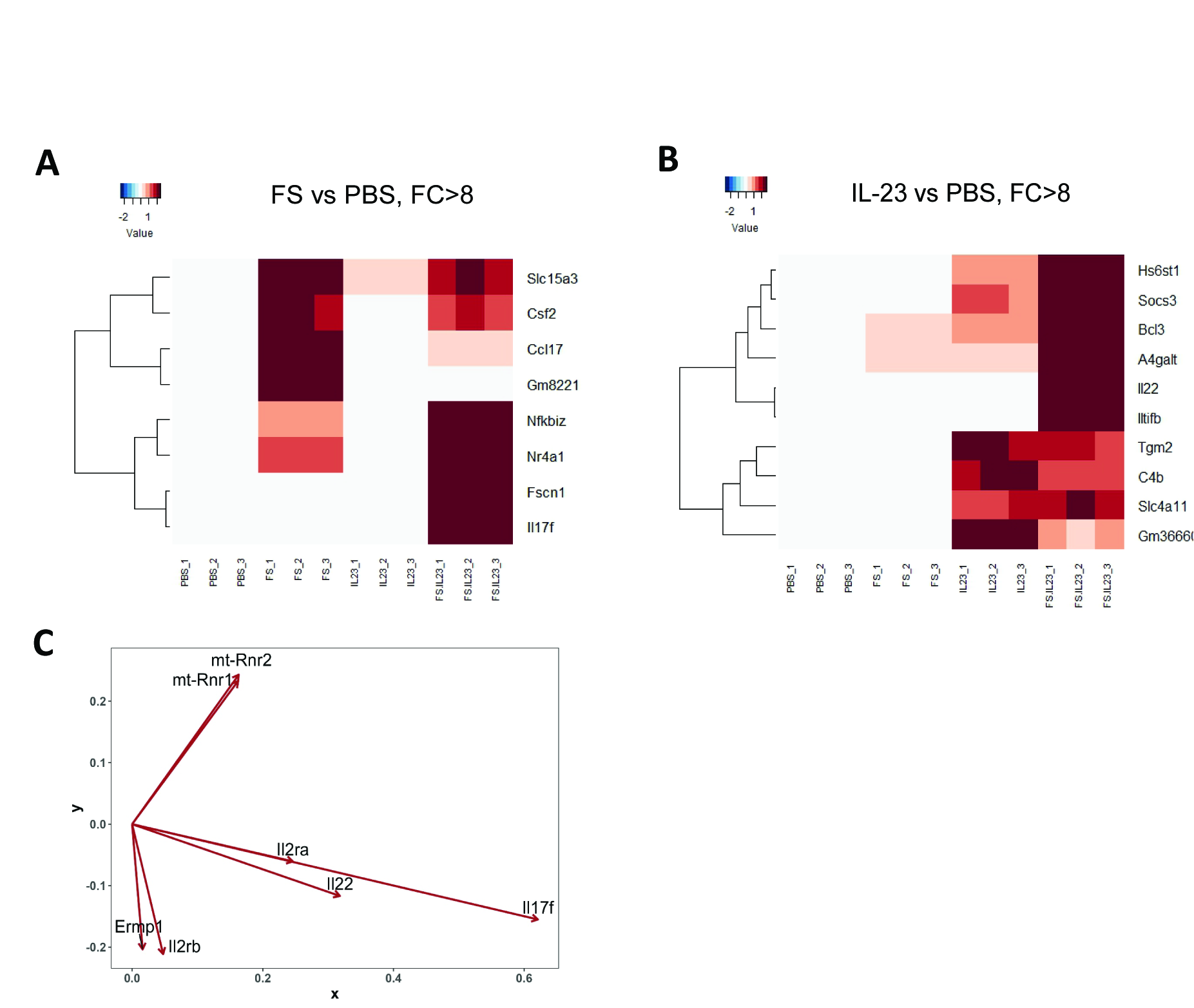

Supplement: S1 Fig — is related to Fig 4. (A and B). Top genes that were differentially expressed by FS (A) and IL-23 (B) compared to the control PBS (FC>8). (C). PCA loading analysis. Il17f, Il22, Il2ra had largest influences on PC1. Mt-Rnr1/2, Il2rb, and Ermp1 had largest influences on PC2. (TIF) [file pone.0317248.s001.tif]

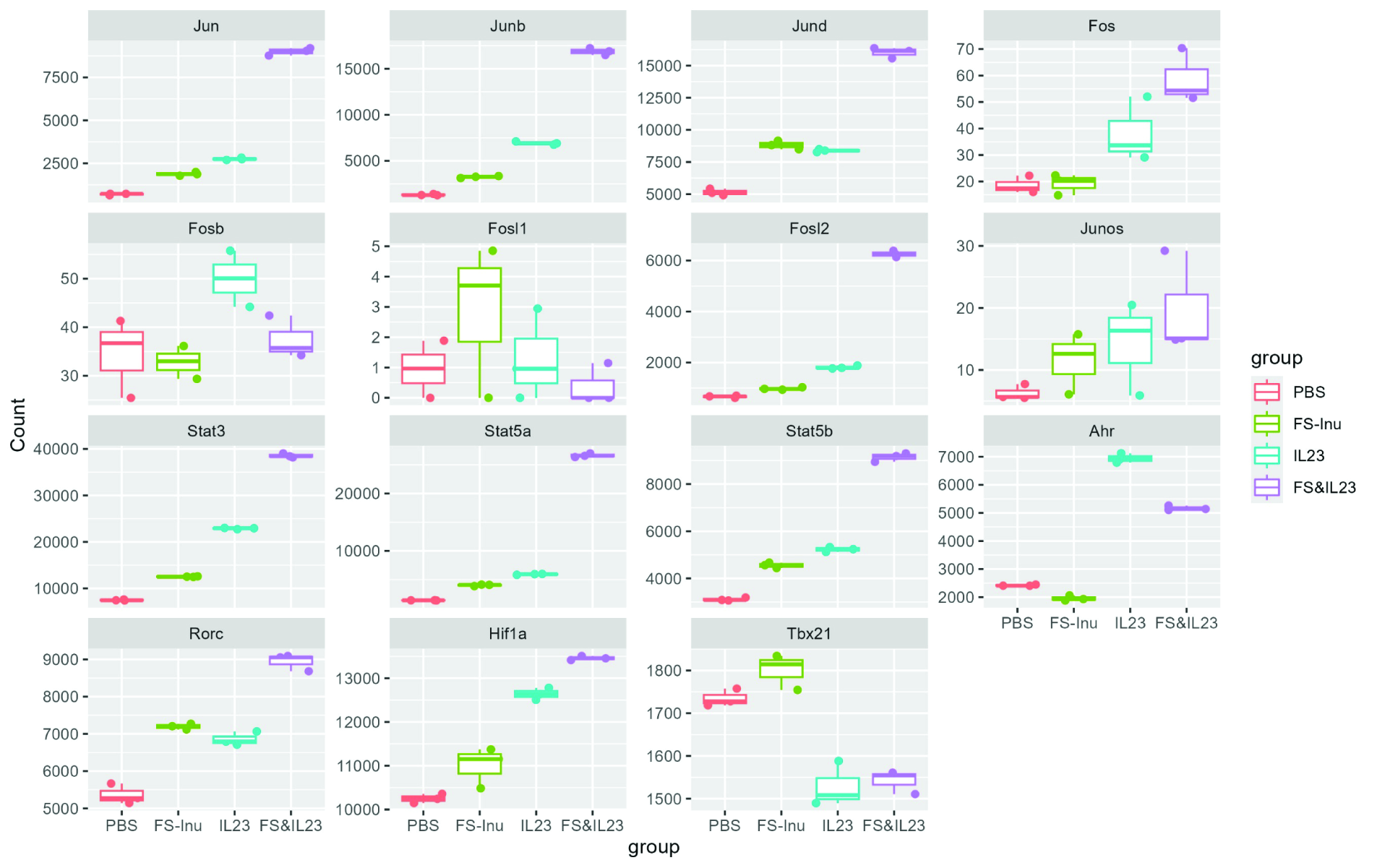

Supplement: S2 Fig — Normalized gene counts data was generated from RNA-seq data. (TIF) [file pone.0317248.s002.tif]

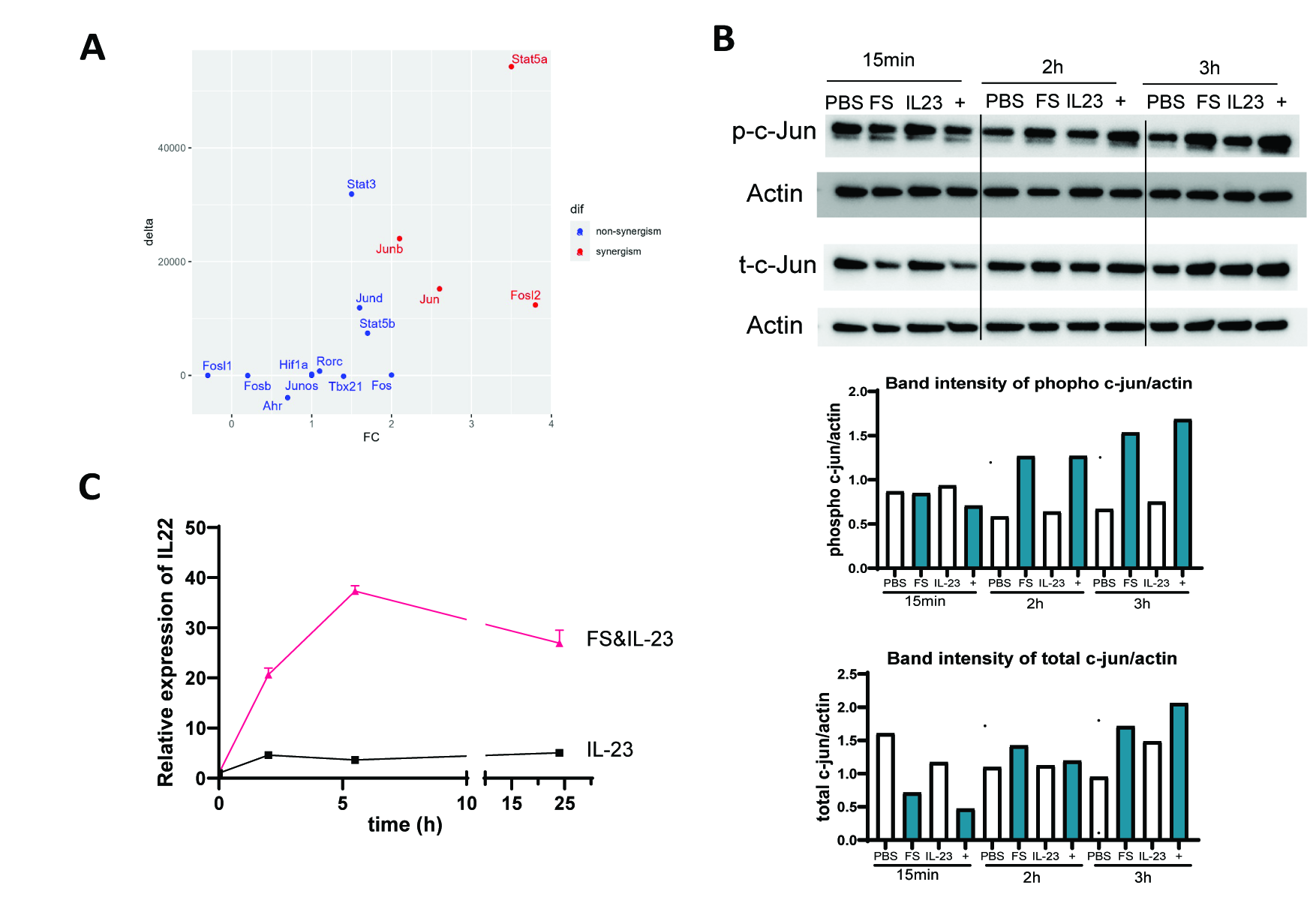

Supplement: S3 Fig — (A) Gene expression of common transcription factors (TF) of IL-22. Normalized gene counts of groups FS, IL23 and FS&IL23 were corrected by the background PBS. FC was the fold change of FS&IL23 to the sum of FS and IL-23 (FS&IL23/(FS+IL23)). Delta was the count difference between FS&IL23 and sum of FS and IL-23 (FS&IL23-(FS+IL23)). Genes were colored red if FC>2.0 and delta>0; otherwise blue. (B) Immunoblotting of c-Jun. Blots of p-c-Jun and t-c-Jun were each stripped and reprobed for actin. Band intensity was quantified by Image Lab (Bio-Rad). (C) IL-22 mRNA expression of MNK-3 cells, treated by IL-23 and FS&IL-23 for indicated time (2 hours, 6 hours, and 24 hours). See raw images and details of S3B an S3C Fig in S1 File. (TIF) [file pone.0317248.s003.tif]
