# Supplementary material for: Microbiota-derived proteins synergize with IL-23 to drive IL22 production in model type 3 innate lymphoid cells
Source: PLoS One. 2025 Jan 13;20(1):e0317248. doi: 10.1371/journal.pone.0317248 (PMC11729933; doi:10.1371/journal.pone.0317248)
Supplement: S1 File — (PDF) [file pone.0317248.s004.pdf]

Original image of Fig 5B (p-stat3 and p-p38)

- This is a merged image from “Chemiluminescence” and “Colorimetric” methods
- Blots of lanes 13-16 were from a separate protein gel due to limited sample lanes in a Bio-Rad mini precast gel, and then combined with gel of lanes 1-12 and transferred together into one blot.
- The blot was cut into two pieces after transfer. Upper blot was then stained with p-stat3 antibody and lower blot was stained with p-p38 antibody. The two blots were taken image together. Exposure time: 2min

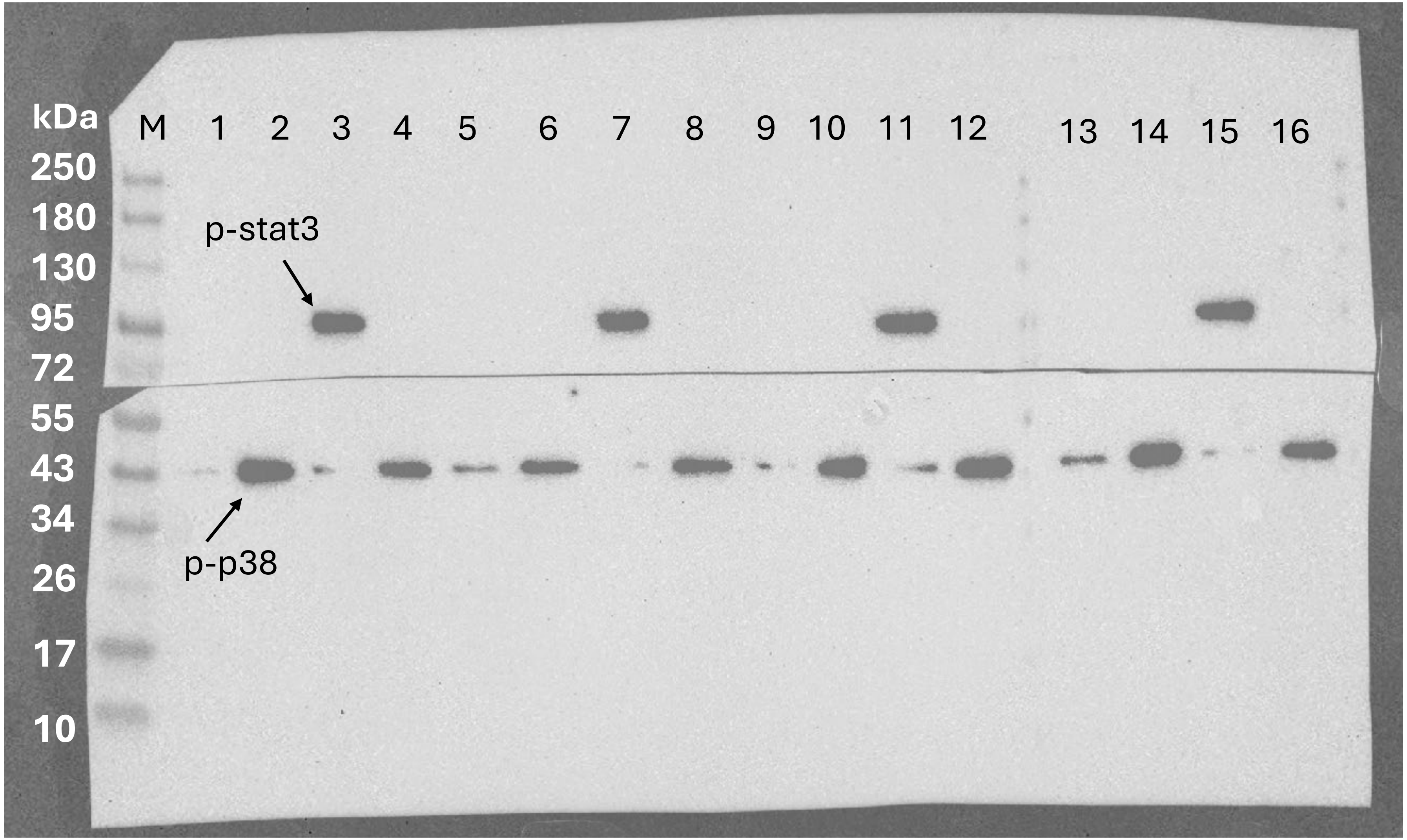

Lane #	sample
1	15min, PBS
2	15min, FS
3	15min, IL-23
4	15min, FS+IL-23
5	1h, PBS
6	1h, FS
7	1h, IL-23
8	1h, FS+IL-23
9	2h, PBS
10	2h, FS
11	2h, IL-23
12	2h, FS+IL-23
13	3h, PBS
14	3h, FS
15	3h, IL-23
16	3h, FS+IL-23

Original image of Fig 5B (t-stat3 and t-p38)

- This is a merged image from "Chemiluminescence" and "Colorimetric" methods
- Blots of lanes 13-16 were from a separate protein gel due to limited sample lanes in a Bio-Rad mini precast gel, and then combined with gel of lanes 1-12 and transferred together into one blot.
- The blot was cut into two pieces after transfer. Upper blot was then stained with t-stat3 antibody and lower blot was stained with t-p38 antibody. The two blots were taken image together. Exposure time: 1min10s

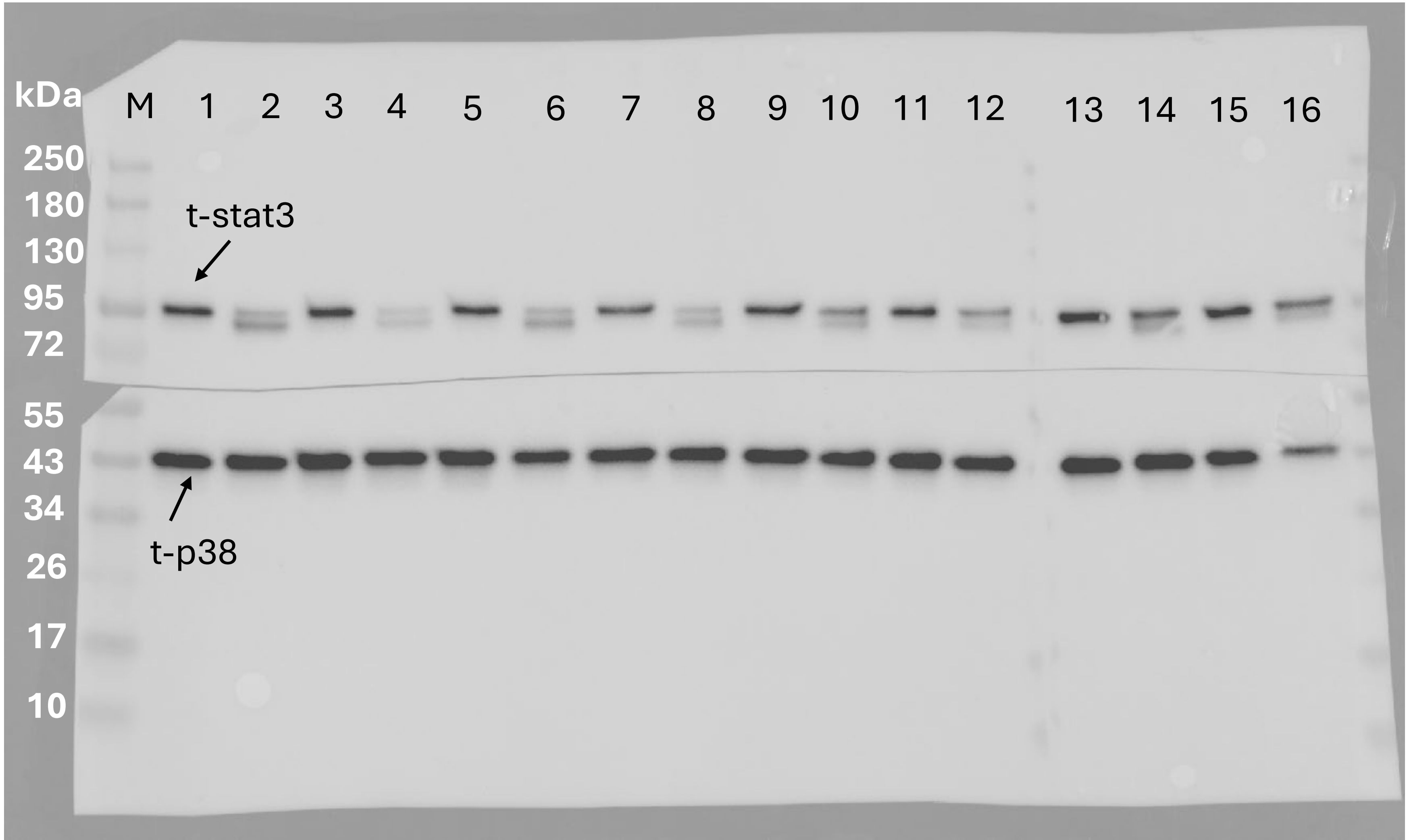

Lane #	sample
M	Marker (NEB P7719S)
1	15min, PBS
2	15min, FS
3	15min, IL-23
4	15min, FS+IL-23
5	1h, PBS
6	1h, FS
7	1h, IL-23
8	1h, FS+IL-23
9	2h, PBS
10	2h, FS
11	2h, IL-23
12	2h, FS+IL-23
13	3h, PBS
14	3h, FS
15	3h, IL-23
16	3h, FS+IL-23

Original image of Fig 5B (actin)

- This is a merged image from "Chemiluminescence" and "Colorimetric" methods
- Blots of lanes 13-16 were from a separate protein gel due to limited sample lanes in a Bio-Rad mini precast gel, and then combined with gel of lanes 1-12 and transferred together into one blot.
- The blot was stained with actin antibody. Exposure time: 2s

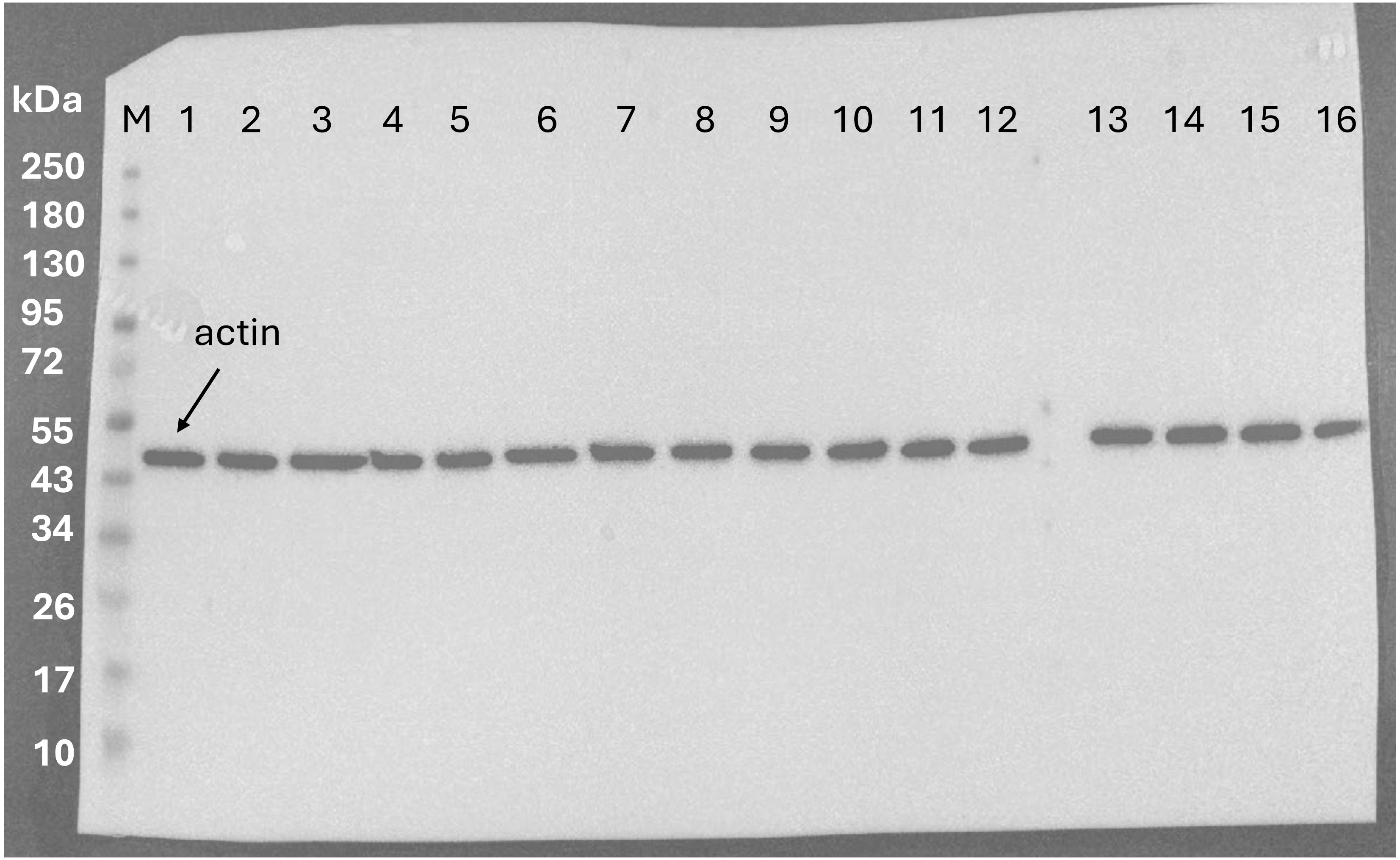

Lane #	sample
M	Marker (NEB P7719S)
1	15min, PBS
2	15min, FS
3	15min, IL-23
4	15min, FS+IL-23
5	1h, PBS
6	1h, FS
7	1h, IL-23
8	1h, FS+IL-23
9	2h, PBS
10	2h, FS
11	2h, IL-23
12	2h, FS+IL-23
13	3h, PBS
14	3h, FS
15	3h, IL-23
16	3h, FS+IL-23

Original image of Fig 5C (p-ErK and actin)

- Each is a merged image from "Chemiluminescence" and "Colorimetric" methods
- The upper blot was stained with p-Erk antibody. Exposure time: 2min.
- The upper blot was then stripped, reblocked, and reprobed with actin antibody (lower blot). Exposure time: 2sec

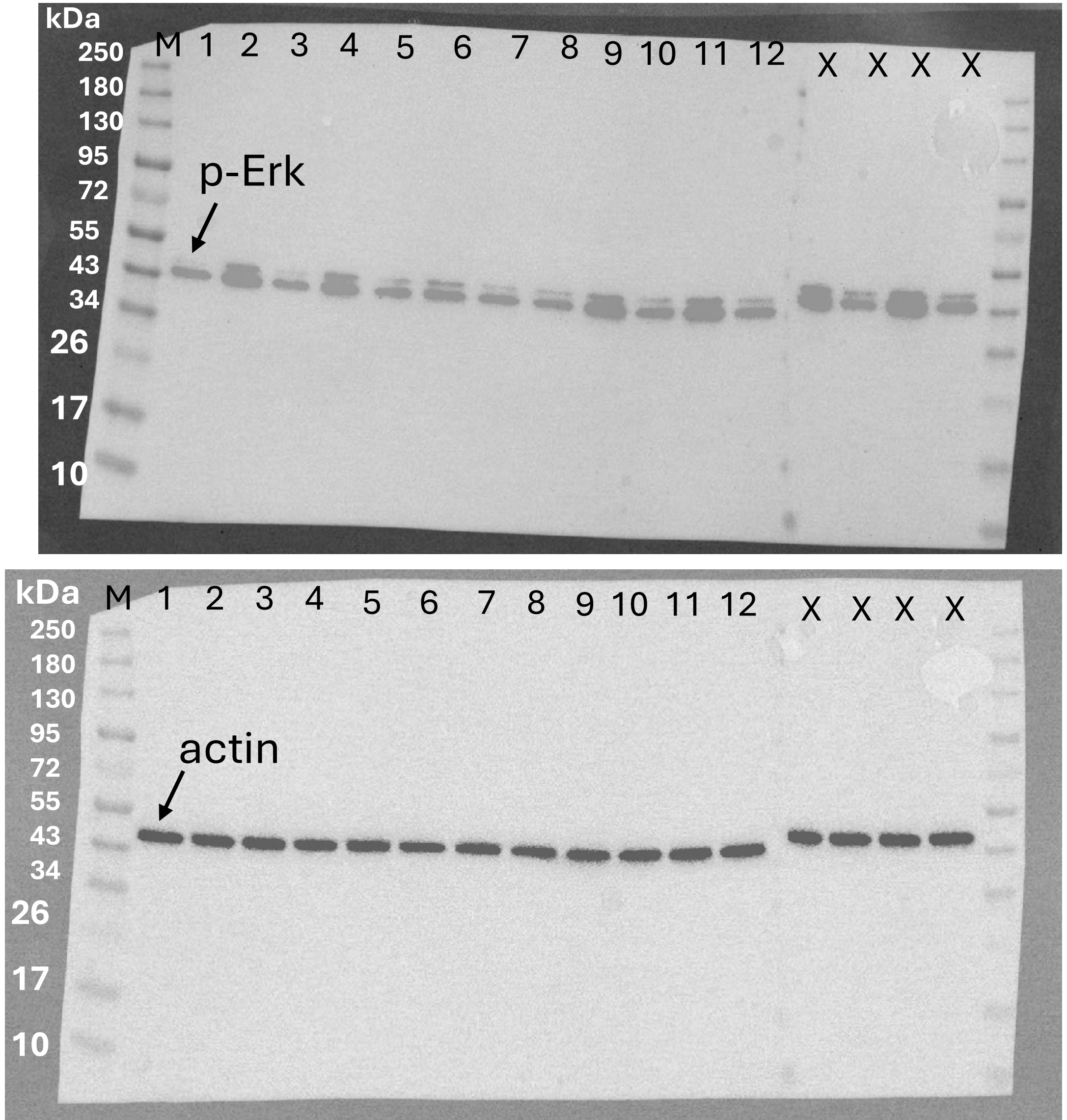

Lane #	sample
M	Marker (NEB P7719S)
1	15min, PBS
2	15min, FS
3	15min, IL-23
4	15min, FS+IL-23
5	1h, PBS
6	1h, FS
7	1h, IL-23
8	1h, FS+IL-23
9	2h, PBS
10	2h, FS
11	2h, IL-23
12	2h, FS+IL-23

Original image of Fig 5C (t-ErK and actin)

- Each is a merged image from “Chemiluminescence” and “Colorimetric” methods
- The upper blot was stained with t-Erk antibody. Exposure time: 2min.
- The upper blot was then stripped, reblocked, and reprobed with actin antibody (lower blot). Exposure time: 2sec

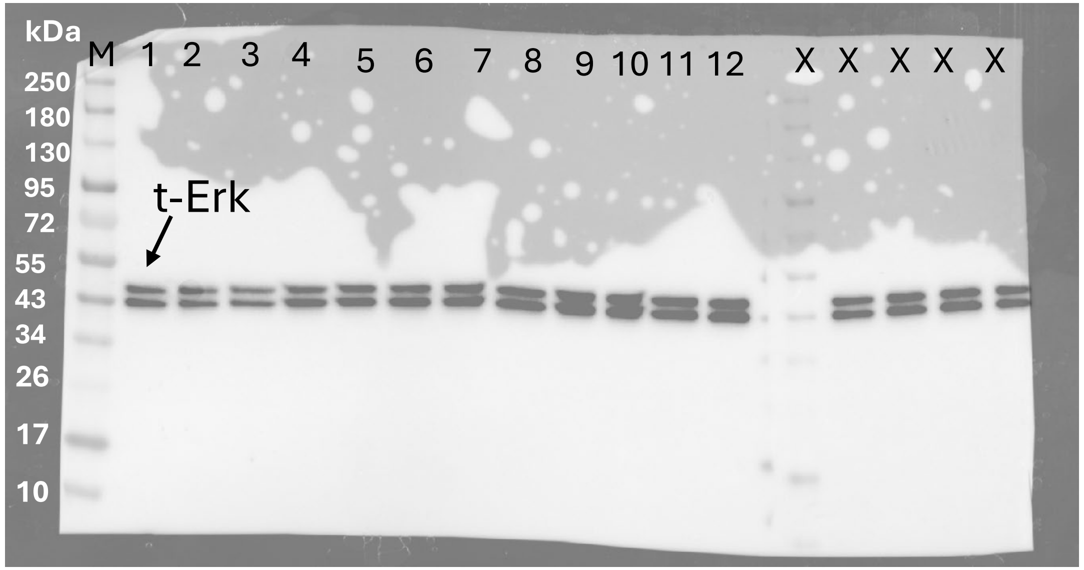

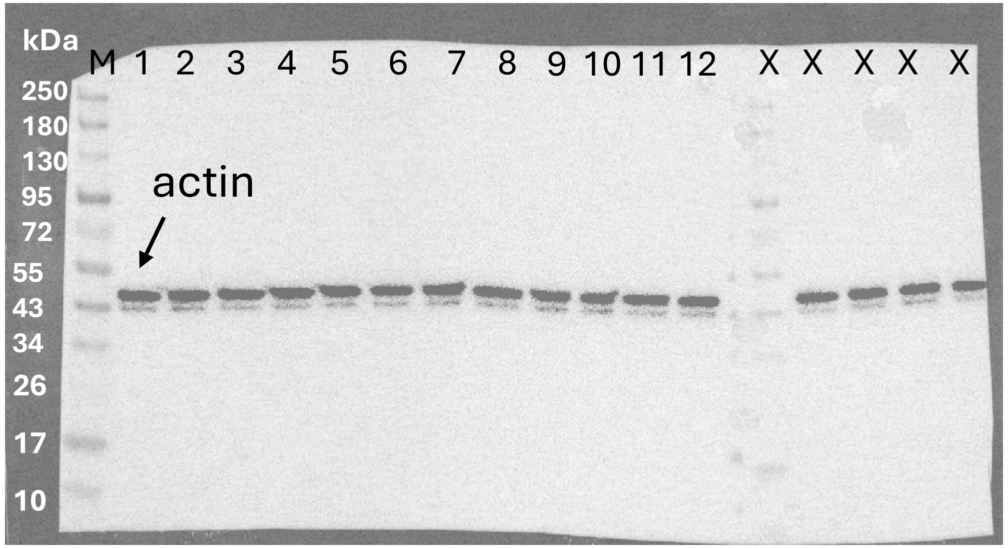

Lane #	sample
M	Marker (NEB P7719S)
1	15min, PBS
2	15min, FS
3	15min, IL-23
4	15min, FS+IL-23
5	1h, PBS
6	1h, FS
7	1h, IL-23
8	1h, FS+IL-23
9	2h, PBS
10	2h, FS
11	2h, IL-23
12	2h, FS+IL-23

Original image of S3B Fig (p-c-Jun and actin)

- Each is a merged image from “Chemiluminescence” and “Colorimetric” methods
- The upper blot was stained with p-c-Jun antibody. Exposure time: 5 min.
- The upper blot was then stripped, reblocked, and reprobed with actin antibody (lower blot). Exposure time: 2sec

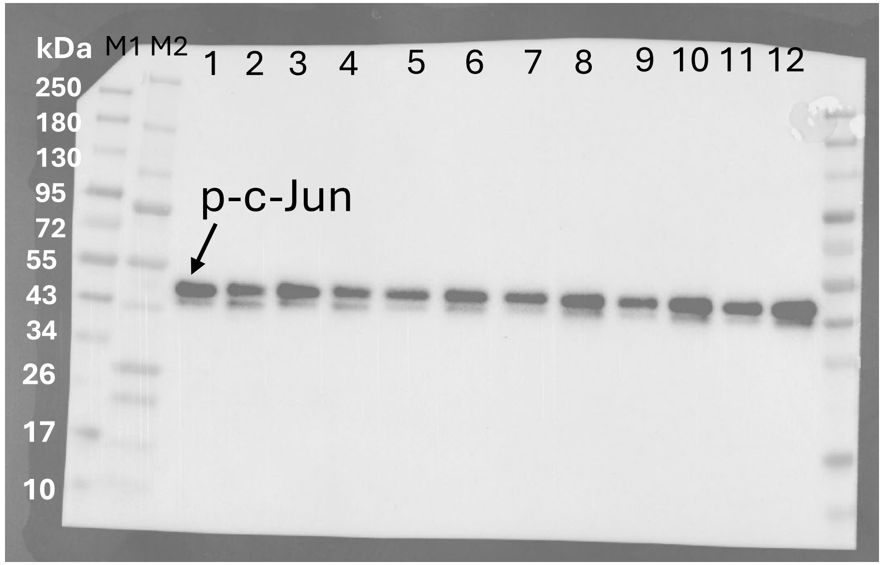

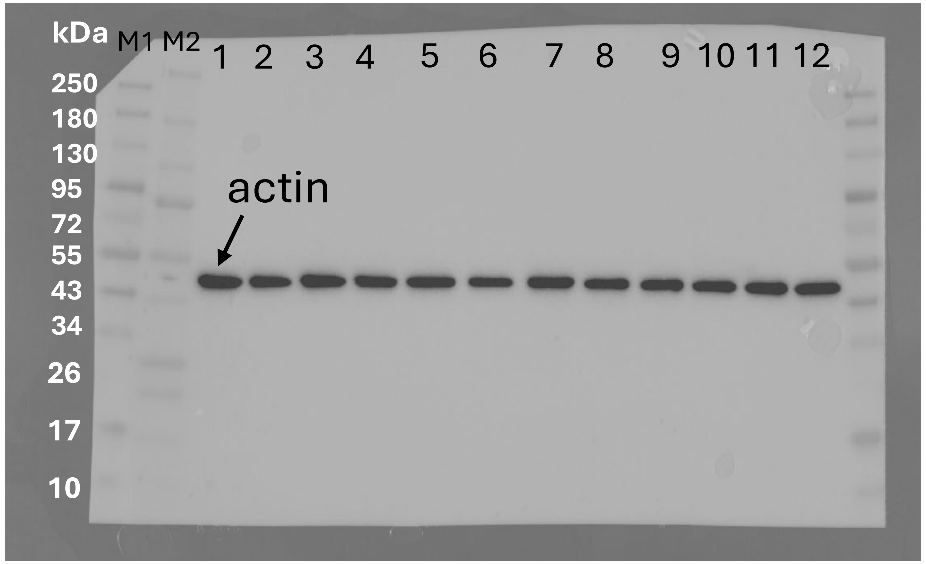

Lane #	sample
M1	Marker (NEB P7719S)
M2	Marker (Biorad #1610363)
1	15min, PBS
2	15min, FS
3	15min, IL-23
4	15min, FS+IL-23
5	2h, PBS
6	2h, FS
7	2h, IL-23
8	2h, FS+IL-23
9	3h, PBS
10	3h, FS
11	3h, IL-23
12	3h, FS+IL-23

Original image of S3B Fig (t-c-Jun and actin)

- Each is a merged image from “Chemiluminescence” and “Colorimetric” methods
- The upper blot was stained with t-c-Jun antibody. Exposure time: 2 min.
- The upper blot was then stripped, reblocked, and reprobed with actin antibody (lower blot). Exposure time: 2sec

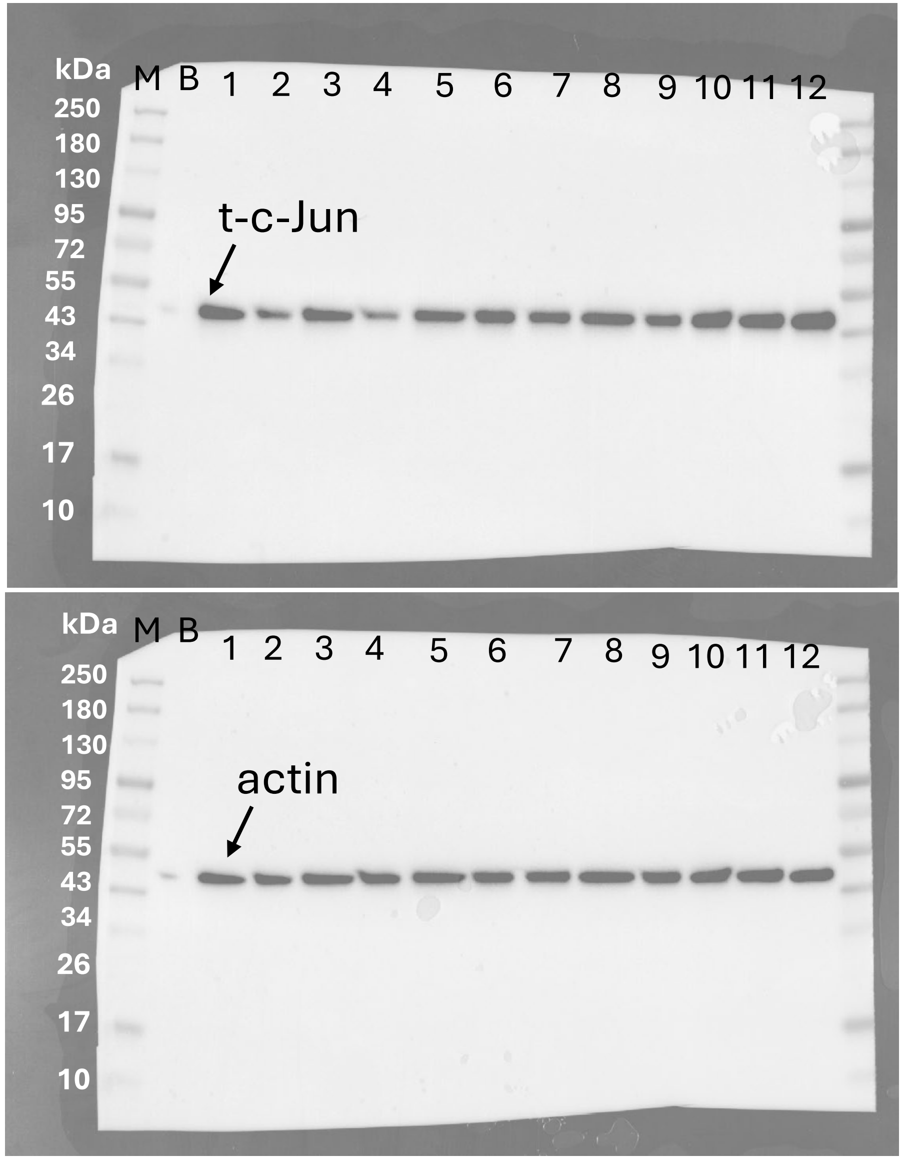

Lane #	sample
M	Marker (NEB P7719S)
B	blank
1	15min, PBS
2	15min, FS
3	15min, IL-23
4	15min, FS+IL-23
5	2h, PBS
6	2h, FS
7	2h, IL-23
8	2h, FS+IL-23
9	3h, PBS
10	3h, FS
11	3h, IL-23
12	3h, FS+IL-23
